# From BIA to BMI: A New Look at Postpartum Recovery and Breastfeeding Outcomes

**DOI:** 10.3390/metabo16010023

**Published:** 2025-12-25

**Authors:** Dominika Mazur, Kornelia Purc-Bandurko, Żaneta Kimber-Trojnar, Marcin Trojnar, Bożena Leszczyńska-Gorzelak

**Affiliations:** 1 Chair and Department of Obstetrics and Perinatology, Medical University of Lublin, 20-090 Lublin, Poland; 2 Chair and Department of Internal Medicine, Medical University of Lublin, 20-081 Lublin, Poland

**Keywords:** bioelectrical impedance analysis, body composition, hydration status, lactation, breastfeeding, postpartum period, maternal nutrition, mode of delivery

## Abstract

**Background/Objectives:** Successful and sustained breastfeeding depends on maternal, psychological, metabolic and obstetric factors including hydration status, body composition, gestational age at delivery and mode of delivery, which are rarely assessed together in routine postpartum care. Bioelectrical impedance analysis (BIA) provides a non-invasive assessment of hydration and tissue composition, yet its potential to support lactation outcomes remains insufficiently studied. This study aimed to evaluate the relationship between postpartum body composition, hydration status assessed with BIA, and breastfeeding duration. **Methods:** A total of 122 women in the early postpartum period after term singleton deliveries were enrolled, of whom 50 completed the full protocol, including a 7-month follow-up. BIA and anthropometric measurements were performed on postpartum days 2 and 3. Breastfeeding duration was assessed at 7 months via telephone interview and categorized as <6 months or ≥6 months. Two indices (PLBI and sPLBI) were calculated to describe BMI change from pre-pregnancy to 7 months postpartum. **Results:** Breastfeeding for ≥6 months was significantly associated with marital status, mode of delivery, lower BMI on postpartum day 2, and a positive change in the overhydration index (ΔOH). Women in this group exhibited significantly lower PLBI and sPLBI values, indicating more effective postpartum weight recovery and a greater return toward pre-pregnancy BMI. Hydration parameters derived from BIA differentiated between shorter and longer breastfeeding duration. **Conclusions:** Positive postpartum hydration balance (ΔOH ≥ 0) and efficient metabolic recovery, reflected by lower PLBI and sPLBI values, may support longer breastfeeding. BIA-based assessment of hydration and body composition could help identify women at higher risk of early breastfeeding cessation. Further longitudinal research is warranted to confirm the clinical utility of BIA in postpartum care and its potential role in early lactation support.

## 1. Introduction

Lactation is a dynamic physiological process requiring adequate maternal nutritional reserves, optimal hydration, stable metabolic balance and appropriate gestational maturity, all of which influence breastfeeding initiation and continuation. The postpartum period is characterized by rapid hormonal shifts, redistribution of body fluids, and changes in tissue composition, which collectively affect both early lactation physiology and maternal recovery. Accurate assessment of maternal physiological status in the early puerperium is therefore important for identifying women at risk of breastfeeding difficulties and for implementing individualized support strategies [1]. Bioelectrical impedance analysis (BIA) offers a non-invasive, rapid, and cost-effective method for evaluating hydration status and body composition; however, its application in postpartum women remains understudied, and existing prediction equations are not tailored to postpartum physiology. Both insufficient hydration and fluid overload have been shown to alter milk production and influence lactation performance, underscoring the importance of objective hydration assessment during the postpartum period. Although BIA is widely used in various clinical and research settings, its specific utility in the postpartum period—particularly in relation to lactation outcomes—remains insufficiently elucidated. Its potential value lies in its ability to provide real-time insights into maternal hydration status and tissue distribution, both of which may directly influence lactation performance [1,2,3]. The recent literature suggests a strong association between maternal body composition and breastfeeding outcomes. Women with unfavorable metabolic or hydration profiles may experience delayed onset of lactation, insufficient milk supply, or early breastfeeding cessation [4]. Conversely, adequate lean body mass and balanced hydration have been linked to improved lactation performance and longer breastfeeding duration [5]. Moreover, maternal hydration is a key determinant of both milk composition and volume, with evidence indicating that both dehydration and fluid overload can negatively affect breastfeeding efficiency [6]. These findings underscore the importance of objective assessment tools, such as BIA, for the early identification of women at risk of suboptimal lactation. Emerging evidence also indicates that the relationship between maternal physiology and lactation is bidirectional. Breastfeeding contributes to the regulation of maternal metabolism, promoting postpartum weight loss, reducing adiposity, and supporting the restoration of metabolic homeostasis [2,7]. It has also been shown to modulate low-grade systemic inflammation, potentially lowering long-term cardiometabolic risk [8]. Notably, exclusive breastfeeding is associated with more favorable postpartum weight trajectories, further emphasizing the relevance of maternal body composition during this period [9].

Despite these promising perspectives, several challenges remain regarding the use of BIA in postpartum populations. Rapid shifts in fluid compartments, breast engorgement, and hormonal fluctuations may affect impedance measurements, and most predictive equations were not developed specifically for postpartum physiology. Device-related variability and methodological inconsistencies—such as differences between single- and multi-frequency measurements, or between whole-body and segmental models—also limit comparability across studies [3,10]. To enhance the clinical applicability of BIA, there is a need for postpartum-specific prediction algorithms and standardized measurement protocols, as well as validation against reference methods. Taken together, current evidence supports the potential of BIA as a valuable tool in postpartum care. By enabling the detection of hydration disturbances, changes in lean mass, and shifts in adiposity, this technique may contribute to more individualized strategies for lactation support. A deeper understanding of the relationship between maternal body composition and lactation physiology may ultimately improve breastfeeding outcomes and support enhanced maternal recovery [11,12,13].

Therefore, the aim of this study was to evaluate the relationship between postpartum body composition and hydration status—measured via BIA twice within 24 h during the early puerperium, prior to hospital discharge—and the duration of breastfeeding, assessed at seven months postpartum. Additionally, the study sought to identify selected sociodemographic and obstetric factors that may influence breastfeeding duration.

## 2. Materials and Methods

This was a prospective, cross-sectional observational study conducted between October 2022 and December 2023 at the Department of Obstetrics and Perinatology in Lublin, Poland, with methodological refinements addressing reviewer comments. Of the initial cohort of 122 participants, 50 completed the full study protocol, including the 7-month follow-up. All participants were in the early postpartum period following term singleton deliveries.

Inclusion criteria were: age ≥ 18 years, singleton pregnancy, and willingness to participate in follow-up assessments regarding breastfeeding status. Exclusion criteria included: pre-existing chronic conditions affecting hydration or metabolism, preterm birth (<37 weeks of gestation), and contraindications to breastfeeding.

Participation was voluntary and based on written informed consent. A total of 122 healthy mothers who had never breastfed previously were recruited.

On postpartum day 2 trained evaluators conducted standardized anthropometric assessments using calibrated digital scales, a wall-mounted stadiometer, and a non-elastic Glick tape. Waist circumference was measured at the midpoint between the lower rib margin and the iliac crest; hip circumference at the level of the greatest gluteal protuberance. Each measurement was taken twice, with a third performed if discrepancies exceeded 0.5 cm. Abdominal skinfold thickness was measured using calipers at the midpoint between the umbilicus and the anterior superior iliac spine, along the vertical midline of the abdomen. Based on these parameters, body mass index (BMI) and waist-to-hip ratio (WHR) were calculated. BIA was performed using the BCM (Body Composition Monitor; Fresenius Medical Care, Bad Homburg, Germany) following a standardized protocol: participants rested supine for 5–10 min; four electrodes were placed on the same side of the body—hand (metacarpal base, 2–3 cm above radial styloid) and foot (metatarsal base, 2–3 cm above the ankle); metal accessories were removed; limbs were positioned to avoid contact. The following parameters were obtained: fat mass (FM), adipose tissue mass (ATM), fat tissue index (FTI), lean tissue mass (LTM), lean tissue index (LTI), body cellular mass (BCM), total body water (TBW), extracellular water (ECW), intracellular water (ICW), and overhydration index (OH).

Participants completed a structured questionnaire previously reviewed for clarity and content by perinatal health experts and pilot -tested in ten postpartum women; although not fully validated, it demonstrated adequate internal coherence. The author’s questionnaire consisted of 51 items, including single- and multiple-choice questions and table-format items, assessing both quantitative and qualitative aspects of maternal health and behaviors during pregnancy. Special emphasis was placed on identifying factors influencing breastfeeding duration and maternal perception of the role of hydration in lactation. The questionnaire was reviewed for clarity and content validity by experts in perinatal health.

On postpartum day 3, a second BIA measurement was performed under the same conditions. For FM, ATM, FTI, LTM, LTI, BCM, TBW, ECW, ICW, and extracellular-to-intracellular water ratio (E/I) the arithmetic mean of values from days 2 and 3 was used in the analysis. For OH, a delta overhydration value (ΔOH) was calculated as the difference between OH on day 3 and OH on day 2.

At seven months postpartum, a structured telephone interview was conducted to assess breastfeeding practices, dietary habits, weight changes, and general health status. The follow-up questionnaire included 12 items on infant feeding methods, growth percentile changes, access to lactation support, water intake, diet type, meal frequency, return to pre-pregnancy weight, current weight, and medication use. Interviews were conducted by trained staff and lasted approximately 10–15 min.

Based on self-reported breastfeeding duration, participants were divided into two groups: <6 months (*n* = 20) and ≥6 months (*n* = 30) of breastfeeding.

For each mother, two BMI were obtained from medical documentation:

- Pre-pregnancy BMI (BMI_0_)
- Pre-delivery BMI (BMI_d_)

For each mother, two BMI measurements were obtained:

- Postpartum day 2 BMI (BMI_p_)
- Seven-month postpartum BMI (BMI_7m_)

These values were used to derive three changes in BMI relative to pre-pregnancy BMI (BMI_0_), calculated at key time points:

ΔBMI_d_ = BMI_d_ − BMI_0_

ΔBMI_p_ = BMI_p_ − BMI_0_

ΔBMI_7m_ = BMI_7m_ − BMI_0_

Two indices (PLBI, sPLBI) were derived based on BMI trajectories. Their mathematical structure was supported by an exploratory elastic-net regression model evaluated via 10-fold cross-validation, used strictly for variable selection rather than prediction:

- Postpartum Longitudinal Body Index (PLBI):

PLBI = ((BMI_d_ − BMI_p_)/ΔBMI_d_) × (ΔBMI_7m_/ΔBMI_d_)

- Simplified PLBI (sPLBI):

sPLBI = ΔBMI_7m_/(BMI_d_ − BMI_7m_)

Both indices are unitless and reflect relative changes in BMI following pregnancy and childbirth.

**Figure 1:**
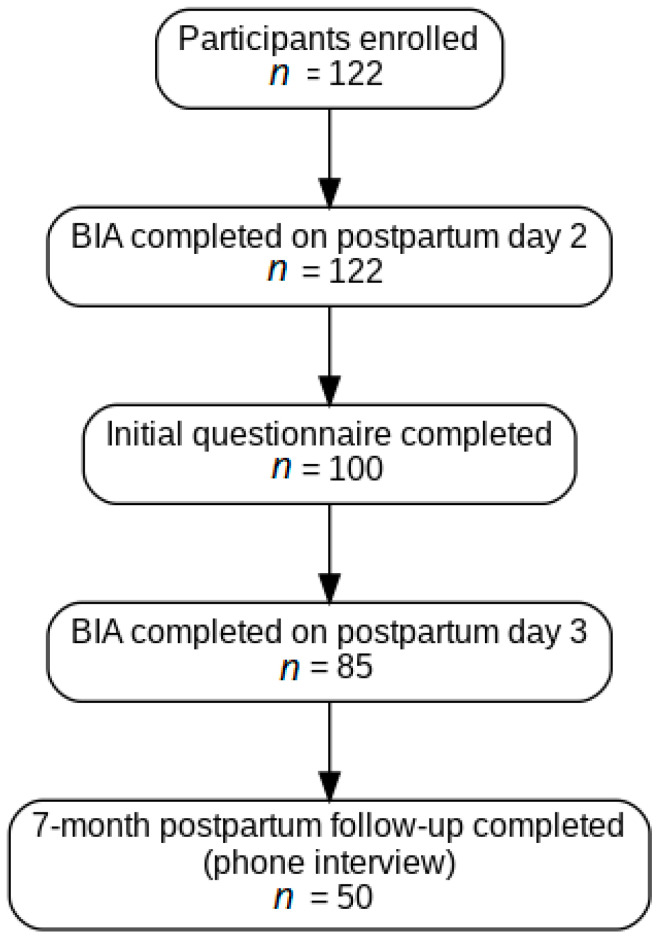
Study Participant Flowchart.

Ultimately, of the 122 women recruited on the day of delivery, complete data from 50 participants were included in the final statistical analysis (Figure 1).

### Statistical Analysis

Data analysis was conducted using Statistica, version 13.3 (TIBCO Software Inc., Palo Alto, CA, USA 1984–2017). Descriptive statistics were calculated for all variables.

Group comparisons were performed using the Mann–Whitney U test for continuous variables and the chi-square test for categorical variables. The Shapiro–Wilk test was used to assess the normality of data distribution.

For variables with a normal distribution, Levene’s test and the F-test were used to assess homogeneity of variance. If assumptions for parametric testing were met, group means were compared using the independent samples Student’s *t*-test.

Correlation analyses were conducted depending on data distribution:

- For normally distributed variables: Pearson’s correlation coefficient
- For non-normally distributed variables: Spearman’s rho

Statistical significance was set at *p* < 0.05. Correlation analyses aimed to identify associations between body composition parameters and breastfeeding duration.

## 3. Results

Among women who breastfed for less than 6 months, the mean duration of breastfeeding was 55.5 days (range: 3 days to 4 months), corresponding to an average of 1.85 ± 1.31 months.

A comparative analysis of sociodemographic and obstetric characteristics was performed between women who breastfed for <6 months and those who continued for ≥6 months with absolute numbers added to frequencies for greater clarity and reproducibility (Table 1).

No statistically significant differences were observed in maternal age or parental educational attainment between the groups. In both groups, the majority of mothers and fathers had tertiary education. Place of residence also did not differ significantly, although urban residence was more common in the ≥6 months group, while a higher proportion of rural residents was noted among those who breastfed for <6 months.

Marital status was significantly associated with breastfeeding duration (*p* = 0.04). A higher proportion of married women was observed in the ≥6 months group, while the <6 months group included more women who were unmarried (single or cohabiting).

Mode of delivery also differed significantly (*p* = 0.001). Vaginal delivery was more prevalent among women who breastfed for ≥6 months, whereas cesarean section predominated in the <6 months group.

Gestational age was significantly higher in the ≥6 months group (*p* = 0.01). Although birth weight was also higher in this group, the difference did not reach statistical significance. No significant differences were observed in Apgar scores or neonatal weight loss on day 3. However, higher Apgar scores (9–10) were more frequent in the ≥6 months group, while lower scores (7–8) were more common in the <6 months group.

No significant differences were found in gestational weight gain between groups.

A comparative analysis of anthropometric parameters between groups is presented in Table 2.

Significant differences were observed in several variables. Pre-pregnancy BMI (BMI_0_) was significantly lower in the <6 months group (*p* = 0.04). However, pre-delivery BMI (BMI_d_) (*p* = 0.002), postpartum BMI on day 2 (BMI_p_) (*p* = 0.002), and BMI at seven months postpartum (BMI_7m_) (*p* = 0.002) were all significantly higher in this group. Similarly, BMI changes from pre-pregnancy to delivery (ΔBMI_d_) were significantly greater among women who breastfed for less than six months.

In contrast, the reduction in BMI between pre-pregnancy and seven months postpartum (ΔBMI_7m_) was significantly greater among women who breastfed for six months or longer, indicating a larger postpartum weight loss in this group (*p* = 0.001).

Adiposity measures, including skinfold thickness (*p* = 0.03), FM (*p* = 0.019), ATM (*p* = 0.018), and FTI (*p* = 0.005), were significantly higher in the <6 months group compared to women who breastfed for ≥6 months. In contrast, no significant differences were found in WHR, LTM, LTI, BCM, or body water distribution (TBW, ECW, ICW, and extracellular-to-intracellular water ratio [E/I]).

Hydration dynamics, assessed by ΔOH, showed a marked contrast: exclusively positive ΔOH values were observed in the ≥6 months group, while negative values predominated in the <6 months group (*p* < 0.001).

Both composite indices, postpartum longitudinal body index (PLBI) and simplified postpartum longitudinal body index (sPLBI), were significantly lower in women who breastfed for 6 months or longer, indicating a more effective postpartum weight reduction and a greater return toward pre-pregnancy BMI in this group (*p* = 0.001 for both).

Anthropometric parameters were correlated with three indices evaluating postpartum body mass changes at seven months: ΔBMI_7m_, PLBI, and sPLBI. These three indicators were analyzed in relation to gestational weight gain, anthropometric measurements taken on postpartum day 2 (including skinfold thickness and waist and hip circumference using Glick’s tape), bioelectrical impedance analysis parameters, four BMI values (pre-pregnancy, pre-delivery, postpartum day 2, and seven months postpartum), as well as BMI changes (deltas) calculated relative to pre-pregnancy BMI (Table 3).

Gestational weight gain showed a moderate positive correlation with ΔBMI_7m_ (*p* = 0.005), PLBI (*p* = 0.016), and sPLBI (*p* = 0.016). No significant correlations were found between skinfold thickness and any of the three indicators.

Positive correlations observed between ΔBMI_7m_ with FM (*p* = 0.02), PLBI with FM (*p* = 0.02), and sPLBI with FM (*p* = 0.014), ΔBMI_7m_ with ATM (*p* = 0.02), PLBI with ATM (*p* = 0.02), and sPLBI with ATM (*p* = 0.016), ΔBMI_7m_ with FTI (*p* = 0.005), PLBI with FTI (*p* = 0.006), and sPLBI with FTI (*p* = 0.003) Meanwhile, LTM and BCM demonstrated negative correlations with ΔBMI_7m_. We observed correlation between LTM with PLBI (*p* = 0.03) and LTM with sPLBI (*p* = 0.04) and between BCM with PLBI (*p* = 0.02) and BCM with sPLBI (*p* = 0.03).

A significant negative correlation was observed between ΔOH and all three indices (ΔBMI_7m_ (*p* = 0.03), PLBI (*p* = 0.05), and sPLBI (*p* = 0.04).

Strong positive correlations were found between BMI_0_, BMI_d_, BMI_p_, and BMI_7m_. Changes in BMI over time—particularly ΔBMI_d_ and ΔBMI_p_—were significantly correlated with ΔBMI_7m_, PLBI, and sPLBI (*p* < 0.001 for all parameters). Notably, ΔBMI_7m_ demonstrated very strong positive correlations with both PLBI (*p* < 0.001) and sPLBI (*p* < 0.001).

**Figure 2:**
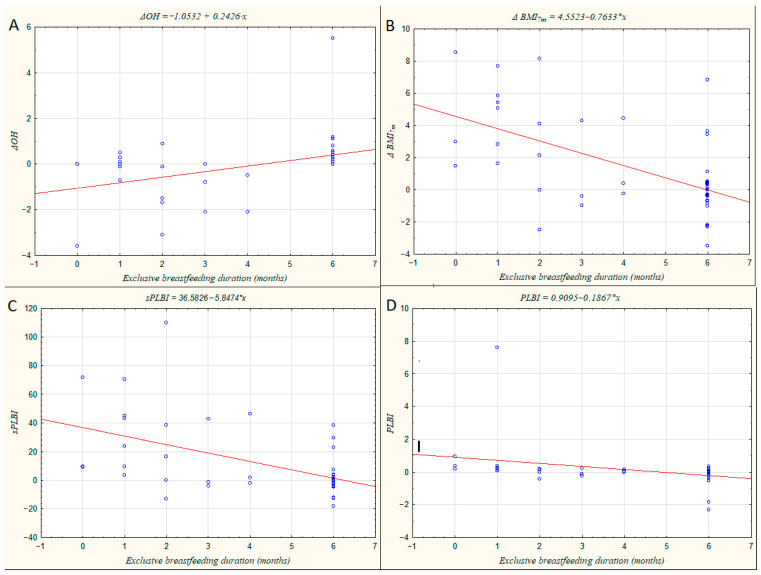
Scatter plots illustrating the relationships between maternal hydration and body composition parameters and the duration of exclusive breastfeeding (in months). (**A**) Change in overhydration index (ΔOH); (**B**) Change in body mass index at seven months postpartum (ΔBMI_7m_); (**C**) Simplified Postpartum Longitudinal Body Index (sPLBI); (**D**) Postpartum Longitudinal Body Index (PLBI).

Figure 2 shows four correlations between the duration of exclusive breastfeeding and selected parameters. A positive association was observed between breastfeeding duration and the change in overhydration index (ΔOH; panel A), with higher ΔOH values predominantly found in women who maintained exclusive breastfeeding for six months or longer. In contrast, the change in BMI at seven months postpartum (ΔBMI_7m_; panel B), as well as sPLBI (panel C) and PLBI (panel D), showed negative correlations with breastfeeding duration. Lower values of these indices were more frequent among women who continued exclusive breastfeeding for ≥6 months, suggesting a more favorable recovery of body mass and composition toward pre-pregnancy levels.

## 4. Discussion

This study investigated the associations between maternal body composition, hydration status, and breastfeeding duration, revealing multiple significant maternal and obstetric factors linked to the continuation of lactation. Our findings emphasize the multi-faceted nature of breastfeeding success, highlighting physiological, psychosocial, and metabolic contributors. However, these findings should be interpreted with caution due to methodological aspects of BIA assessment in the immediate postpartum period, as discussed below. Importantly, the use of BIA non-invasive insights into postpartum recovery dynamics, and the indices introduced in this study—PLBI, sPLBI, and ΔOH—represent exploratory tools that may support individualized postpartum assessment, although they require further validation.

One of the most prominent psychosocial factors identified was marital status. Women who were married demonstrated a significantly higher likelihood of breastfeeding for at least six months compared to their unmarried or cohabiting counterparts. This is consistent with previous research indicating that emotional stability, partner involvement, and reduced perceived stress enhance lactation physiology by supporting oxytocin release and maternal confidence. Married women may benefit from greater emotional stability, partner support, and reduced stress, all of which contribute to increased maternal confidence and commitment to breastfeeding [14,15,16]. Conversely, a lack of partner or family support can create emotional distress, impair hormonal regulation, and ultimately shorten lactation duration, a phenomenon described in the literature as affecting oxytocin-mediated milk ejection and maternal motivation [16,17].

The mode of delivery emerged as a strong predictor of breastfeeding persistence, corroborating existing evidence. Vaginal delivery was significantly associated with longer breastfeeding duration, while cesarean section correlated with early cessation. Cesarean births also involve perioperative intravenous fluids that can temporarily alter hydration measurements, potentially influencing ΔOH values—a factor that should be considered when interpreting BIA-derived hydration parameters. This finding reflects known physiological mechanisms, where cesarean birth can delay the onset of lactogenesis II, disrupt early skin-to-skin contact, and interfere with the hormonal cascade essential for milk production and ejection, including oxytocin and prolactin release [18,19,20]. These disturbances may lead to difficulties in establishing breastfeeding, which have been documented in multiple cohorts worldwide [19]. Our data support the importance of facilitating vaginal birth when medically feasible and encouraging early breastfeeding initiation post-cesarean to mitigate these effects. Although our results remained consistent after taking delivery mode into account, this confounder warrants attention in future research.

Gestational age was another critical factor, with longer pregnancies favoring sustained lactation. Infants born closer to term are likely to have more mature suckling reflexes and greater physiological readiness to breastfeed, which facilitates effective milk transfer and enhances maternal confidence. This aligns with previous studies that have linked prematurity with breastfeeding difficulties and earlier cessation. More mature neonatal feeding reflexes and physiological readiness at birth likely contribute to this effect, aligning with prior studies [21,22,23]. Although birth weight did not reach statistical significance in our study, trends suggested that higher birth weights were observed among infants of mothers who breastfed for longer durations, possibly reflecting better neonatal health and more efficient feeding.

Maternal anthropometric measures provided crucial insight into postpartum adaptation and metabolic recovery. Notably, higher BMI values immediately postpartum and at seven months were characteristic of women who discontinued breastfeeding earlier. Furthermore, the extent of BMI change from pre-pregnancy to seven months postpartum (ΔBMI_7m_) was significantly greater in those who maintained breastfeeding ≥6 months, indicating more efficient postpartum weight loss. These patterns are consistent with research showing that lactation promotes energy expenditure and fat mobilization [24,25,26,27,28,29,30]. PLBI and sPLBI strongly correlated with ΔBMI_7m_, supporting their potential relevance but also underscoring the need for external validation before clinical use.

Adiposity-related parameters, including skinfold thickness, FM, ATM, and FTI, were consistently higher in women who ceased breastfeeding earlier. Excess adiposity may impair lactation through hormonal imbalances such as insulin resistance and decreased prolactin sensitivity, factors implicated in lactation physiology disruption [31,32,33,34,35]. Conversely, lean body mass measures—LTM and BCM—negatively correlated with PLBI and sPLBI, suggesting that greater lean mass supports energy balance and metabolic flexibility necessary for sustained milk production. This highlights the importance of maintaining or improving lean tissue during the postpartum period as a modifiable factor potentially influencing breastfeeding outcomes [36,37,38,39,40,41].

A particularly novel and clinically relevant finding concerns hydration status assessed via ΔOH. Women who breastfed for ≥6 months exhibited exclusively positive ΔOH values, indicating an increase or normalization in fluid balance postpartum. In contrast, those who stopped breastfeeding earlier showed predominantly negative ΔOH values, suggesting impaired fluid regulation or incomplete recovery of extracellular water homeostasis. The negative correlations between ΔOH and indices such as ΔBMI_7m_, PLBI, and sPLBI imply that proper hydration and resolution of pregnancy-related fluid retention accompany effective metabolic recovery and lactation persistence. The normalization of extracellular water and plasma volume likely facilitates adequate mammary gland perfusion, essential for milk synthesis and ejection [41,42,43]. Thus, ΔOH emerges as a promising, non-invasive marker of maternal recovery with potential clinical application in routine postpartum monitoring. However, ΔOH must be interpreted with caution: BIA may be influenced by engorgement, rapid postpartum fluid shifts, and perioperative fluids. The lack of hydration diaries limits interpretation of ΔOH and should be addressed in future studies.

Gestational weight gain also demonstrated positive correlations with ΔBMI_7m_, PLBI, and sPLBI, suggesting that excessive weight gain during pregnancy predisposes women to persistent adiposity and may indirectly contribute to earlier breastfeeding cessation. These findings echo existing recommendations advocating for controlled gestational weight gain to optimize maternal and neonatal health outcomes [43,44,45]. Monitoring weight trajectories during pregnancy and providing tailored nutritional counseling could mitigate adverse postpartum metabolic profiles and support breastfeeding.

The integrated pattern of physiological and psychosocial variables in our cohort highlights the complexity of breastfeeding determinants. Favorable profiles for sustained breastfeeding included vaginal delivery, longer gestation, marital stability, lower postpartum BMI, reduced adiposity, greater lean mass, and positive ΔOH. Conversely, cesarean delivery, excessive gestational weight gain, higher postpartum BMI, and negative hydration patterns were linked with premature breastfeeding cessation. These multifactorial influences emphasize the need for comprehensive postpartum care addressing both physical recovery and psychosocial support.

The use of BIA and its derived indices, including those introduced in our study—PLBI and sPLBI—provides clinicians with a practical approach for the objective monitoring of postpartum recovery. These indices may help identify women at risk of delayed metabolic normalization or premature breastfeeding cessation, enabling timely interventions such as nutritional counseling, lactation support, and hydration management [17,18,45,46]. Changes in body composition play a significant role in milk production. Incorporating BIA into standard postpartum care could enhance individualized support and improve breastfeeding outcomes.

The strengths of this study include its prospective design, standardized BIA measurements (conducted twice, 24 h apart during the early postpartum period), and a seven-month follow-up, which allowed for longitudinal assessment of maternal recovery. However, several limitations should be acknowledged. The reliance on self-reported data regarding breastfeeding duration and feeding practices introduces potential bias and inconsistencies. Psychosocial factors beyond marital status—such as perceived stress, emotional well-being, and broader social support networks—were not evaluated but likely play an important role in lactation outcomes. Furthermore, the relative cultural and socioeconomic homogeneity of the study population limits the generalizability of these findings to more diverse groups, where differing environmental and social contexts may influence breastfeeding continuation [43,44,45,46,47].

In summary, our findings highlight a complex interplay of physiological and psychosocial factors influencing breastfeeding persistence. Vaginal delivery, longer gestation, marital stability, lower postpartum adiposity, greater lean mass, and improved hydration status (ΔOH) collectively support sustained lactation. Novel BIA-derived indices like PLBI and ΔOH represent promising tools for postpartum monitoring and individualized care. Further research in larger, heterogeneous populations is warranted to validate these markers and optimize breastfeeding support strategies. Although PLBI, sPLBI, and ΔOH appear promising as tools for postpartum monitoring, they should be considered exploratory and require further validation before clinical application [45,46,47,48].

## 5. Conclusions

Breastfeeding duration beyond six months appears to be influenced by an integrated set of obstetric, anthropometric, hydration-related, and psychosocial factors.

Vaginal delivery, higher gestational age, marital stability, lower postpartum adiposity, and positive ΔOH values supported sustained lactation. Conversely, cesarean section, excessive gestational weight gain, higher early postpartum BMI, and negative hydration trends were associated with earlier cessation.

The Postpartum Longitudinal Body Index (PLBI) and its simplified version PLBI (sPLBI) emerged as informative indicators of postpartum metabolic recovery, providing a greater interpretive value than ΔBMI_7m_ alone. However, these indices should be considered exploratory and require external validation before they can be applied clinically.

BIA may support breastfeeding management by identifying clinically meaningful hydration changes—particularly ΔOH—that reflect physiological recovery during the first postpartum days. Targeted professional education for midwives and postpartum staff about hydration physiology and BIA interpretation could support more effective breastfeeding counseling prior to hospital discharge. Nevertheless, the clinical utility of BIA-based markers must be confirmed in larger, more diverse populations with standardized measurement protocols.

Incorporating BIA-derived hydration markers, especially ΔOH into. Early postpartum care may help identify women at heightened risk of early breastfeeding cessation and guide individualized lactation and nutritional interventions. PLBI and sPLBI may offer additional value in assessing metabolic recovery trajectories and tailoring postpartum support strategies, though further validation in larger and more diverse populations is warranted.

## Figures and Tables

**Table 1:** Sociodemographic and obstetric characteristics of the study population by breastfeeding duration (<6 months vs. ≥6 months).

Variables		<6-Month Group	≥6 Month Group	*p*
Age [years] Median (Range)		31 (22–39)	30 (25–47)	0.23
Mother’s education [%]	Primary	0	3	0.31
	Lower secondary	0	3	
	Vocational secondary	15	3	
	General secondary	20	17	
	Tertiary	65	74	
Father’s education [%]	Primary	5	7	0.77
	Lower secondary	5	0	
	Vocational secondary	20	10	
	General secondary	20	13	
	Tertiary	50	70	
Place of residence [%]	Large city (>500 k residents)	5	13	0.14
	Medium city (100–500 k residents)	30	43	
	Small city (50–100 k residents)	15	7	
	Town (10–50 k residents)	15	17	
	Village	35	20	
Marital status [%]	Married	75	97	0.04
	Unmarried–single	10	3	
	Unmarried–cohabiting	15	0	
Mode of delivery [%]	vaginal delivery	5	53	0.001
	cesarean section	95	47	
Gestational age [weeks] Median (Range)		37.4 (34–41)	38.77 (33–42)	0.01
Birth weight [g] Median (Range)		3006 (1940–3880)	3326 (1860–3870)	0.06
Apgar score [points]	7	5	3.33	0.21
	8	10	3.33	
	9	10	33.33	
	10	75	60	
Physiological neonatal weight loss on day 3 [%] Median (Range)		6.95 (0–16)	5.57 (2–11)	0.21
Gestational weight gain [kg] Median (Range)		13.8 (1–35)	13 (3–22)	0.76

**Table 2:** Comparison of anthropometric characteristics of the study population according to breastfeeding duration (<6 months vs. ≥6 months).

Variables		<6-Month Group M ± SD	≥6 Month Group M ± SD	*p*
BMI_0_ (kg/m^2^)		24.84 ± 3.9	25.12 ± 3.35	0.04
BMI_d_ (kg/m^2^)		31.795 ± 4.08	28.197 ± 3.4	0.002
ΔBMI_d_ (kg/m^2^)	≥0 (%)	100	100	not applicable
	<0 (%)	0	0	
Skinfold thickness (cm)		18.3 ± 6.82	14.2 ± 6	0.03
WHR		0.838 ± 0.05	0.83 ± 0.05	0.55
FM (kg)		33.105 ± 8.67	27.187 ± 8.26	0.019
ATM (kg)		45.035 ± 11.80	36.947 ± 11.28	0.018
FTI (kg/m^2^)		17.58 ± 6.96	13.14 ± 3.37	0.005
LTM (kg)		33.42 ± 9.23	32.953 ± 12.06	0.96
LTI (kg/m^2^)		12.6 ± 4.41	11.853 ± 4.13	0.59
BCM (kg)		16.89 ± 5.24	17.37 ± 8.58	1.0
TBW (L)		33.265 ± 5.34	31.547 ± 6.82	0.18
ECW (L)		15.110 ± 2.5	14.653 ± 2.54	0.53
ICW (L)		17.66 ± 3.64	16.907 ± 4.74	0.3
E/I		0.899 ± 0.12	0.884 ± 0.12	0.38
ΔOH	<0 (%)	65	0	<0.001
	≥0 (%)	35	100	
BMI_p_ (kg/m^2^)		28.215 ± 3.79	25.123 ± 2.86	0.002
ΔBMI_p_ (kg/m^2^)	≥0 (%)	86.67	95	0.14
	<0 (%)	13.33	5	
BMI_7m_ (kg/m^2^)		27.88 ± 4.6	22.77 ± 3.08	0.002
ΔBMI_7m_ (kg/m^2^)	≥0 (%)	46.67	75	0.001
	<0 (%)	53.33	25	
PLBI		0.52 ± 1.69	−0.18 ± 0.55	0.001
sPLBI		26.02 ± 31.85	1.33 ± 11.26	0.001

ATM—adipose tissue mass; BCM—body cell mass; BMI_0_—pre-pregnancy body mass index; BMI_d_—pre-delivery body mass index; BMI_p_—body mass index on postpartum day 2; BMI_7m_—body mass index at seven months postpartum; ΔBMI_d_—change in BMI from pre-pregnancy to delivery; ΔBMI_p_—change in BMI from pre-pregnancy to postpartum day 2; ΔBMI_7m_—change in BMI from pre-pregnancy to seven months postpartum; ΔOH—change in overhydration index (day 3 minus day 2); E/I—extracellular-to-intracellular water ratio; ECW—extracellular water; FM—fat mass; FTI—fat tissue index; ICW—intracellular water; LTI—lean tissue index; LTM—lean tissue mass; PLBI—postpartum longitudinal body index; sPLBI—simplified postpartum longitudinal body index; TBW—total body water; WHR—waist-to-hip ratio.

**Table 3:** Spearman’s rank correlation coefficients between gestational weight gain, body composition parameters, and BMI indices.

Variable	Δ BMI_7m_	PLBI	sPLBI
Gestational weight gain	*r* = 0.39; *p* = 0.005	*r* = 0.34; *p* = 0.016	*r* = 0.34; *p* = 0.016
Skinfold thickness	*r* = 0.002; *p* = 0.99	*r* = −0.02; *p* = 0.87	*r* = 0.01; *p* = 0.94
ΔOH	*r = −0.3; p = 0.03*	*r = −0.28; p = 0.05*	*r = −0.3; p = 0.04*
FM	*r* = 0.32; *p* = 0.02	*r* = 0.32; *p* = 0.02	*r* = 0.34; *p* = 0.014
ATM	*r* = 0.32; *p* = 0.02	*r* = 0.31; *p* = 0.02	*r* = 0.34; *p* = 0.016
FTI	*r* = 0.39; *p* = 0.005	*r* = 0.39; *p* = 0.006	*r* = 0.41; *p* = 0.003
LTM	*r* = −0.24; *p* = 0.09	*r* = −0.30; *p* = 0.03	*r* = −0.29; *p* = 0.04
BCM	*r* = −0.25; *p* = 0.08	*r* = −0.32; *p* = 0.02	*r* = −0.30; *p* = 0.03
TBW	*r* = −0.1; *p* = 0.49	*r* = −0.13; *p* = 0.36	*r* = −0.10; *p* = 0.48
ECW	*r* = −0.01; *p* = 0.93	*r* = −0.03; *p* = 0.82	*r* = −0.01; *p* = 0.93
E/I	*r* = 0.05; *p* = 0.75	*r* = 0.13; *p* = 0.39	*r* = 0.10; *p* = 0.49
BMI_0_	*r* = −0.22; *p* = 0.13	*r* = −0.19; *p* = 0.19	*r* = −0.17; *p* = 0.23
BMI_d_	*r* = 0.11; *p* = 0.45	*r* = 0.10; *p* = 0.48	*r* = 0.12; *p* = 0.42
BMI_p_	*r* = 0.14; *p* = 0.35	*r* = 0.13; *p* = 0.36	*r* = 0.15; *p* = 0.29
ΔBMI_d_	*r* = 0.51; *p* < 0.001	*r* = 0.45; *p* < 0.001	*r* = 0.43; *p* = 0.002
ΔBMI_p_	*r* = 0.65; *p* < 0.001	*r* = 0.53; *p* < 0.001	*r* = 0.58; *p* < 0.001
ΔBMI_7m_	-	*r* = 0.95; *p* < 0.001	*r* = 0.97; *p* < 0.001

ATM—adipose tissue mass; BCM—body cell mass; BMI_0_—pre-pregnancy body mass index; BMI_d_—pre-delivery body mass index; BMI_p_—body mass index on postpartum day 2; BMI_7m_—body mass index at seven months postpartum; ΔBMI_d_—change in BMI from pre-pregnancy to delivery; ΔBMI_p_—change in BMI from pre-pregnancy to postpartum day 2; ΔBMI_7m_—change in BMI from pre-pregnancy to seven months postpartum; ΔOH—change in overhydration index (day 3 minus day 2); E/I—extracellular-to-intracellular water ratio; ECW—extracellular water; FM—fat mass; FTI—fat tissue index; ICW—intracellular water; LTI—lean tissue index; LTM—lean tissue mass; PLBI—postpartum longitudinal body index; sPLBI—simplified postpartum longitudinal body index; TBW—total body water; WHR—waist-to-hip ratio.

## Data Availability

The data presented in this study are available on request from the corresponding author.

## Abbreviations

The following abbreviations are used in this manuscript:

ATM	adipose tissue mass
BCM	body cell mass
BMI_0_	pre-pregnancy body mass index
BMI_d_	pre-delivery body mass index
BMI_p_	body mass index on postpartum day 2
BMI_7m_	body mass index at seven months postpartum
ΔBMI_d_	change in BMI from pre-pregnancy to delivery
ΔBMI_p_	change in BMI from pre-pregnancy to postpartum day 2
ΔBMI_7m_	change in BMI from pre-pregnancy to seven months postpartum
ΔOH	change in overhydration index (day 3 minus day 2)
E/I	extracellular-to-intracellular water ratio
ECW	extracellular water
FM	fat mass
FTI	fat tissue index
ICW	intracellular water
LTI	lean tissue index
LTM	lean tissue mass
PLBI	postpartum longitudinal body index
sPLBI	simplified postpartum longitudinal body index
TBW	total body water
WHR	waist-to-hip ratio
